# Ginsenoside Rg5 Inhibits Succinate-Associated Lipolysis in Adipose Tissue and Prevents Muscle Insulin Resistance

**DOI:** 10.3389/fphar.2017.00043

**Published:** 2017-02-14

**Authors:** Na Xiao, Le-Le Yang, Yi-Lin Yang, Li-Wei Liu, Jia Li, Baolin Liu, Kang Liu, Lian-Wen Qi, Ping Li

**Affiliations:** State Key Laboratory of Natural Medicines, China Pharmaceutical UniversityNanjing, China

**Keywords:** Ginsenoside Rg5, hypoxia, ER stress, succinate, Lipolysis, insulin resistance

## Abstract

Endoplasmic reticulum (ER) stress, inflammation, and lipolysis occur simultaneously in adipose dysfunction and contribute to insulin resistance. This study was designed to investigate whether ginsenoside Rg5 could ameliorate adipose dysfunction and prevent muscle insulin resistance. Short-term high-fat diet (HFD) feeding induced hypoxia with ER stress in adipose tissue, leading to succinate accumulation due to the reversal of succinate dehydrogenase (SDH) activity. Rg5 treatment reduced cellular energy charge, suppressed ER stress and then prevented succinate accumulation in adipose tissue. Succinate promoted IL-1β production through NLRP3 inflammasome activation and then increased cAMP accumulation by impairing PDE3B expression, leading to increased lipolysis. Ginsenoside Rg5 treatment suppressed NLRP3 inflammasome activation, preserved PDE3B expression and then reduced cAMP accumulation, contributing to inhibition of lipolysis. Adipose lipolysis increased FFAs trafficking from adipose tissue to muscle. Rg5 reduced diacylglycerol (DAG) and ceramides accumulation, inhibited protein kinase Cθ translocation, and prevented insulin resistance in muscle. In conclusion, succinate accumulation in hypoxic adipose tissue acts as a metabolic signaling to link ER stress, inflammation and cAMP/PKA activation, contributing to lipolysis and insulin resistance. These findings establish a previously unrecognized role of ginsenosides in the regulation of lipid and glucose homeostasis and suggest that adipose succinate-associated NLRP3 inflammasome activation might be targeted therapeutically to prevent lipolysis and insulin resistance.

## Introduction

Adipose tissue functions as a site for fat storage, whereas disordered fat storage and mobilization are tightly associated with insulin resistance and diabetes. The elevated level of plasma free fatty acids (FFAs) is often observed in individuals subject to insulin resistance and diabetes (Coppack et al., 1992). The abnormal fat deposit has increased lipolytic ability, and therefore, enhanced delivery of FFAs to the liver or muscle, leading to ectopic lipid deposition and insulin resistance.

It is generally accepted that adipose hypoxia is a result of increased fat mass size (Skurk et al., 2007; Trayhurn, 2013; Muniyappa and Sowers, 2014). However, a recent study demonstrates that saturated fatty acids induce adipose hypoxia through increasing oxygen consumption in adipocytes without alternation in the fat mass size (Lee et al., 2014). This finding sheds new insight into the impact of lipid challenge in adipose dysfunction. As a cellular stress, hypoxia induces endoplasmic reticulum (ER) stress in adipose tissue. Aside being a center for protein folding, the ER functions as a major signal-transducing organelle to sense cellular homeostasis. Cellular stress activates transmembrane sensors, including inositol-requiring enzyme 1α (IRE1α), RNA-dependent protein kinase-like ER kinase (PERK) and activating transcription factor 6 (ATF6) to induce unfolded-protein response or ER stress. ER stress is proposed to trigger oxidative stress, inflammation and cell death in specialized cells or tissues, and recent findings show that ER stress augments lipolysis in adipose tissue (Deng et al., 2012; Bogdanovic et al., 2015). Although hypoxia, ER stress, and lipolysis simultaneously occur in adipose dysfunction (Kawasaki et al., 2012; Trayhurn, 2013), the mechanisms and unifying framework for these events are not yet fully understood.

As an important intermediate of the mitochondrial citric acid cycle (CAC), succinate emerges as a metabolic signaling mediator which is responsible for the adaptation of tissues to hypoxic conditions. The conventional direction of succinate dehydrogenase (SDH) in the CAC is to drive succinate toward fumarate formation. However in ischemic heart, succinate accumulation arises from reversal of SDH activity, a process partially relative with the malate/aspartate shuttle (Chouchani et al., 2014). As succinate accumulation induces ROS production and tissue damage in ischemic heart, and increases hypoxia-inducible factor-1α (HIF-1α) induction and IL-1β expression in macrophages (Tannahill et al., 2013), it is tempting to know if succinate acts as a metabolic signaling in hypoxic adipose tissue dysfunction.

Ginseng root has been used for thousands of years in traditional Chinese medicine. Ginsenosides are the major bioactive constituents in ginseng root with beneficial effects on the regulation of glucose and lipid metabolism (Shang et al., 2008; Lee H. M. et al., 2012; Yuan et al., 2012). Ginsenoside Rg5 is the most abundant compound in steamed ginseng (Qi et al., 2011) and its action in the regulation of metabolic homeostasis is unknown. This work was designed to investigate the effects of Rg5 on the regulation of lipolysis in hypoxic adipose tissue with emphasis on the regulation of succinate accumulation and the second messenger cAMP. Herein, we indicate that succinate accumulated in the adipose tissue of high-fat diet (HFD) fed mice, linking hypoxia and ER stress with lipolysis through NLRP3 inflammasome activation. Ginsenoside Rg5 inhibited succinate-associated lipolysis by reducing cellular energy charge, and thus ameliorated insulin resistance in the muscle by reducing lipid deposits. These findings provide novel insights into the effect of Rg5 on the regulation of glucose and lipid homeostasis.

## Materials and methods

### Reagents

Rg5 (purity > 95%) was purchased from Jiangsu Yongjian Pharmaceutical Technology Co., Ltd. (Jiangsu, China). Tauroursodeoxycholic Acid (TUDCA), dimethyl succinate, dimethyl malonate, thapsigargin (TG), isobutylmethylxanthine (IBMX), dexamethasone, insulin, and Ro-20-1724 were obtained from Sigma (St Louis, MO, USA), while forskolin was from Abcam Cambridge, USA. Palmitate (PA, Sinopharm, Shanghai, China) was dissolved in ethanol to prepare 200 mM stock solution and then diluted with medium containing 10% FFA-free BSA at the ratio of 1:19 before use. The following items were purchased from the cited commercial sources: anti-HIF-1α antibody (PA1-16601) from Thermo Scientific; anti-IL-1β antibody from R&D; anti-PERK (#3192), anti-phospho-(Ser/Thr) PKA substrate (#9621), anti-phospho-HSL (Ser660) (#4126), anti-HSL (#4107), anti-NLRP3 (#15101), and anti-phospho-Akt (Thr308) (#8205) from Cell Signaling Technology (Beverly, MA, USA); anti-phospho-IRE1α (S724) (ab104157), anti-IRE1α (ab37073), and anti-cleaved caspase-1/anti-caspase-1 (ab1872) from Abcam; anti-phospho-PERK (Thr981) (sc-32577) and anti-PDE3B (sc-20793) from Santa Cruz; anti-Akt (A444), GADPH and Goat Anti-Rabbit IgG (H+L) HRP from Bioworld Technology (St. Paul, MN, USA); Ciolostamide (B6500) from ApexBio

### Cell culture and differentiation

3T3-L1 (a cell line of preadipocyte, from ATCC) were cultured in Dulbecco's Minimum Essential Medium (DMEM, Gibco, USA) supplemented with 10% FBS, 100 μg/mL of streptomycin and 100 U/mL of penicillin at 37°C in a 5% CO_2_ atmosphere. The cells were treated when grew to 80–90% confluence with DMEM containing 0.5 mM IBMX, 1 mM dexamethasone, 10 μg/mL insulin for 2 days. The cells were then incubated in DMEM (10% FBS) containing 10 μg/mL insulin alone for another 2 days. Adipocytes were used 8–10 days after the initiation of differentiation.

C2C12 mouse myoblasts obtained from the Type Culture Collection of the Chinese Academy of Sciences (Shanghai, China), were cultured in DMEM with 10% FBS. C2C12 myoblasts were grown to 80–90% confluence and then induced to differentiate by changing the medium from 10% FBS to 2% horse serum and left for up to 5 days. The medium was changed every 2 days during the entire experimental period.

### Animals

Male ICR mice (18–20 g) were supplied by the Laboratory Animal Center of Nanjing Qinglongshan and were housed in colony cages with 12:12 h light/dark cycles. All experiments and animal care were conducted in accordance with the Provision and General Recommendation of Chinese Experimental Animals Administration Legislation and were approved by Animal Ethics Committee of China Pharmaceutical University. For HFD study, mice were fed with HFD (10% Lard, 10% yolk, 1% cholesterol, 0.2% cholate and 78.8% standard diet) for 10 days simultaneously with administration of Rg5 or TUDCA (50 mg/kg) by oral gavage every day. After being fasted, blood was collected from the orbital sinus for biochemical assay using commercial kits (Jiancheng, Nanjing, China).

### Preparation of conditioned medium (CM)

The fat located in the epididymis from HFD fed mice was isolated and chopped into small pieces and 100 mg weight taken. The prepared adipose tissue was incubated in 2 mL DMEM or KRH buffer (Krebs-Ringer phosphate- HEPES buffer, containing 118-mM NaCl, 5-mM KCl, 1.3-mM CaCl_2_, 1.2-mM MgSO_4_, 1.2-mM KH_2_PO_4_, and 30-mM HEPES, containing 0.5% BSA, pH 7.4) supplemented with 10% FBS for 16 h. Then, the supernatants were collected by centrifugation as CM and stored at −70°C.

### Oral glucose load test

After being fasted overnight, HFD-fed mice were administered glucose solution (2 g/kg) orally. For CM challenge, normal mice were fasted overnight and injected with CM (i.p. 0.1 mL /10 g) for 30 min before glucose load. Blood samples were collected from the orbital sinus at regular intervals after glucose load, serum glucose was tested with a commercial kit based on the glucose oxidase peroxidase (GOD-POD) method. AUC was calculated for blood glucose as the following formula: 0.5 × [Bg0 + Bg0.5]/2 + 0.5 × [Bg0.5 + Bg1.0]/2 + 1 × [Bg1.0 + Bg2.0]/2 (Bg0, Bg0.5, Bg1.0, and Bg2.0 referred to the blood glucose concentration at 0, 0.5, 1.0, and 2.0 h after the glucose load).

### Pimonidazole staining for hypoxia

To detect hypoxia staining in adipose tissue, HFD-fed mice were intraperitoneally injected with pimonidazole (60 mg/kg) (Hypoxyprobe-1 plus kit, Hypoxyprobe, Inc., Burlington, USA) prior to sacrifice. Adipose tissue located in the epididymis was removed and fixed in 4% paraformaldehyde. The isolated adipose tissue was dehydrated and embedded in paraffin and then cut into thickness and affixed onto the slides. The slides were immunostained with FITC-conjugated antibodies (1:100) against pimonidazole adducts according to the manufacturer's instructions. For hypoxia detection in adipocytes, pimonidazole (200 μM) was added into cells with the indicated agents, and then incubated with palmitate (PA, 100 μM) for 8 h or exposed to hypoxia (1% O_2_) for 4 h. Cells were fixed with 4% paraformaldehyde and permeabilized with 0.3% Triton X-100 for 10 min and then blocked with 5% BSA for 1 h. After stained with FITC-conjugated antibodies for 1 h at 37°C, cells were washed three times with PBS. The image was visualized using fluorescence microscope.

### Detection of mitochondrial complex I and IV activity

Differentiated adipocytes were cultured in 15 cm diameter plates and treated with Rg5 (10 μM) or metformin (1 mM) for 4 h, and the cells were collected after PBS wash. Mitochondria were isolated by Mitochondria Isolation Kit (Jiancheng, Nanjing, China). Then, 10 μg of protein was collected for the assay of complex I or IV activity with commercial kit (Jiancheng, Nanjing, China). For ATP measurement, 3T3-L1 cells were treated with regents for 4 h, the cells extracted with lysis buffer, and centrifuged at 12 000 g for 15 min at 4°C. After quantification, the supernatants were detected by ATP Assay Kit (Beyotime, Nanjing, China).

### Oxygen consumption ratio (OCR) measurements

OCR measurement was done using the XFe96 Extracellular Flux Analyzer (Seahorse Bioscience, North Billerica, MA, USA). Differentiated adipocytes were seeded (10^4^ cells/well) and incubated overnight in DMEM supplemented with 10% FBS. The cells were treated with Rg5 (10 μM) or metformin (1 mM) for 4 h. One hour before the measurement, the cells were incubated with XF medium at 37°C in a CO_2_-free incubator. The OCR was detected under basal condition and after the application of 1 μM oligomycin, 0.5 μM FCCP (carbonyl cyanide p-trifluoromethoxyphenylhydrazone) and 0.5 μM rotenone + 0.5 μM antimycin A (XF Cell Q17 Mito Stress Test Kit; Seahorse Bioscience). OCR result was analyzed using the Seahorse XF96 software. Each sample was analyzed four times.

### Mitochondrial ROS analysis

Adipocytes were pretreated with Rg5 (10 μM), TUDCA (100 μM), rotenone (1 μM) with or without PA (100 μM) for 8 h, and then incubated with MitoSox Red (5 μM, Molecular Probes, Eugene, OR) and Mito-Tracker Green (30 nM, Beyotime, Shanghai, China) for 30 min. Labeled cells were analyzed by confocal microscopy.

### Succinate quantification

Differentiated adipocytes (10^6^) stimulated with PA or TG (1 μM) for 8 h or 10 mg adipose tissue of HFD-fed mice were rapidly homogenized on ice in 100 μL of ice-cold succinate assay buffer. Centrifuge at 10,000 g for 5 min to collect the supernatant. The supernatant was measured by succinate colorimetric assay kit (Sigma, St Louis, USA) following the manufacturer's instructions.

### Phosphodiesterases (PDEs) and succinate dehydrogenase (SDH) activity in adipocytes

Differentiated adipocytes were stimulated with PA or dimethyl succinate at given concentrations for 8 h. Cells were collected by centrifugation. After washing with cold PBS, resuspend cells in PBS were subjected to ultrasonication for four times, and then centrifuged to remove cellular debris. The protein concentration was determined by Bicinchoninic Acid Protein Assay kit. The activity was measured by succinate dehydrogenase assay kit (Jiancheng, Nanjing, China) or PDE activity assay kit (Abcam, Cambridge, USA).

### Measurement of glycerol, free fatty acids, IL-6, TNF-α, and IL-1β

*In vitro*, differentiated adipocytes were cultured in serum-free DMEM, and pretreated with indicated agents in the presence of PA (100 μM) for 2 h. After washing, the cells were cultured for another 6 h. *In vivo*, HFD-fed mice were sacrificed by cervical dislocation. The epididymis adipose tissue was chopped into small pieces and 100 mg weight taken to and incubated in 2 mL DMEM for 16 h. The supernatants were analyzed for glycerol (Jiancheng, Nanjing, China) and free fatty acids (calvin, Suzhou, China) by commercial kits. IL-6, TNF-α and IL-1β in the medium was determined using ELISA (Cusabio Biotech, Wuhan, China).

### cAMP, AMP, diacylglycerol (DAG) and ceramides quantification

Differentiated adipocytes were collected, subjected to ultrasonication and centrifuged to remove cellular debris. Adipose tissues were rinsed and weighed before homogenization. After homogenization, the resulting suspension was subjected to ultrasonication and centrifugation. The contents of cAMP (USCN, Wuhan, China) AMP (Chengbin Biotech, Shanghai, China) in the supernatant were measured by ELISA following the manufacturer's instructions.

For HFD-fed mice, skeletal muscle was removed, weighed and then homogenized in 0.9% NaCl (1: 9, *v/v*). For normal mice, after treatment with CM (*i.p*. 1 ml/10 g) for 1 h, the skeletal muscle was isolated and homogenized. After centrifugation, the supernatant was measured for DAG and ceramides contents using commercial kits (Calvin, Suzhou. China).

### Immunohistochemistry for HSL

Differentiated adipocytes were stimulated with PA (100 μM) for 8 h fixed in 4% paraformaldehyde for 15 min, and then blocked with 5% BSA containing 0.3% Triton X-100 for 1 h. The cells were incubated with primary antibodies (1:100) overnight at 4°C and probed with anti-Rabbit IgG-FITC for 1 h in the dark. Immunofluorescent HSL signal was observed under a Nikon Eclipse Ti-s microscope.

### Detection of 2-NBDG uptake by C2C12 cells with fluorescence microscopy

After serum starvation, differentiated C2C12 cells were stimulated with CM in KRH buffer for 30 min and then incubated with 500 μM 2-NBDG (Invitrogen, Oregon, USA) containing 100 nM insulin for another 30 min. Glucose uptake was observed under fluorescence microscope.

### Western blot analysis

Tissue or cell proteins were extracted with lysis buffer, the lysates were centrifuged and the supernatants were collected. For the assay of membrane PKCθ, membrane protein was prepared with plasma membrane protein extraction kit (KeyGEN Biotech, Nanjing, China). The supernatants were quantified with Bicinchoninic Acid Protein Assay kit (Biosky Biotechnology Corporation, Nanjing, China). After quantification, 40 μg protein samples were resolved by 10% SDS-PAGE and transferred electrophoretically to PVDF membranes, blocked, and then incubated with primary antibodies (1:1000) and secondary antibody (1:1000). The antibody reactivity was then detected by ECL and quantified by densitometry with IPP 6.0 software. The assessment of HSL and JNK phosphorylation was performed at the same time for the similar molecular weight of GAPDH (36 kDa) and JNK (46 kDa). The protein expression levels of GAPDH, HSL, and JNK were separately detected, and then HSL and JNK phosphorylation were simultaneously detected on one gel with consistent experimental conditions.

### Statistics

Results were expressed as mean ± SD. For comparison between the two groups, Student's *t*-test was used. To compare more than two groups, statistical analysis was performed by one-way ANOVA followed by the Student's *t*-test. Values of *p* < 0.05 were considered statistically significant.

## Results

### Rg5 attenuated hypoxia in adipose tissue

**Figure 1:**
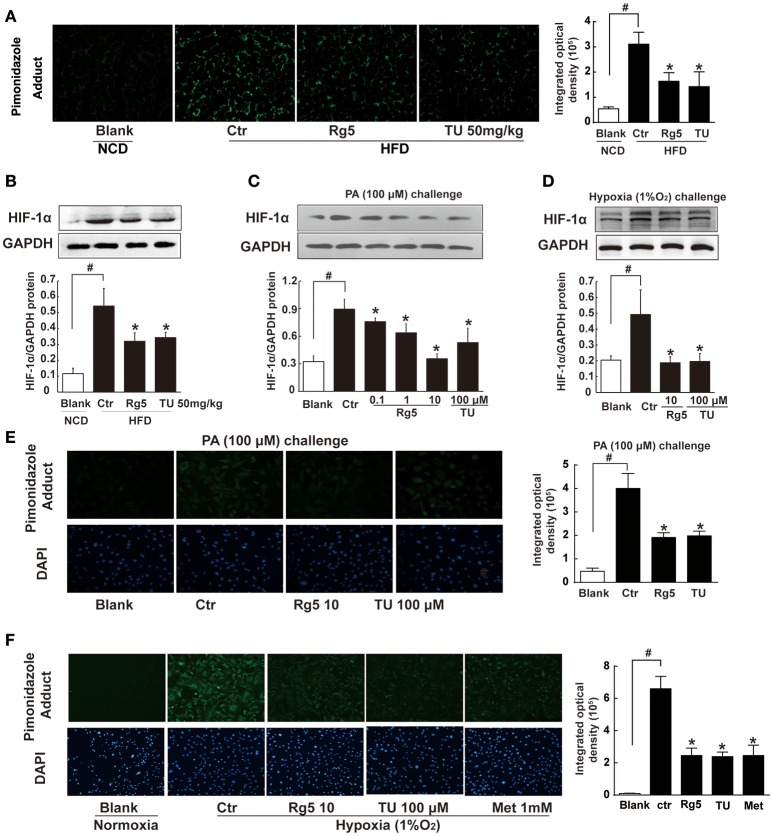
**Effects of Rg5 on adipose hypoxia. (A)** Adipose hypoxia examination with pimonidazole staining in HFD mice. **(B)** HIF-1α protein expression in adipose tissue of HFD-fed mice. **(C,D)** HIF-1α protein expression in differentiated adipocytes treated with palmitate (PA), 1% O_2_ for 8 h. **(E,F)** Pimonidazole staining in differentiated adipocytes treated with PA for 8 h or 1% O_2_ for 4 h. The results were expressed as the mean ± SD of three independent experiments. ^*^p < 0.05 *vs*. Control (Ctr), ^#^*p* < 0.05 *vs*. Blank. TU, Tauroursodeoxycholic Acid; Met, Metformin.

Short term-HFD feeding (10 days) in mice induced hypoxia in adipose tissue, indicated by increased pimonidazole staining and HIF-1α accumulation, whereas these alterations were prevented by Rg5 or ER stress inhibitor TUDCA administration (Figures 1A,B). Rg5 and TUDCA also inhibited HIF-1α induction in adipocytes exposed to PA or hypoxia (1% O_2_) treatment (Figures 1C,D). Meanwhile, Rg5 and TUDCA treatment prevented low oxygen tension in adipocytes (Figures 1E,F), suggesting that Rg5 inhibited HIF-1α accumulation by preventing lower oxygen tension.

### Rg5 attenuated hypoxia in adipocytes by reducing cellular energy charge

**Figure 2:**
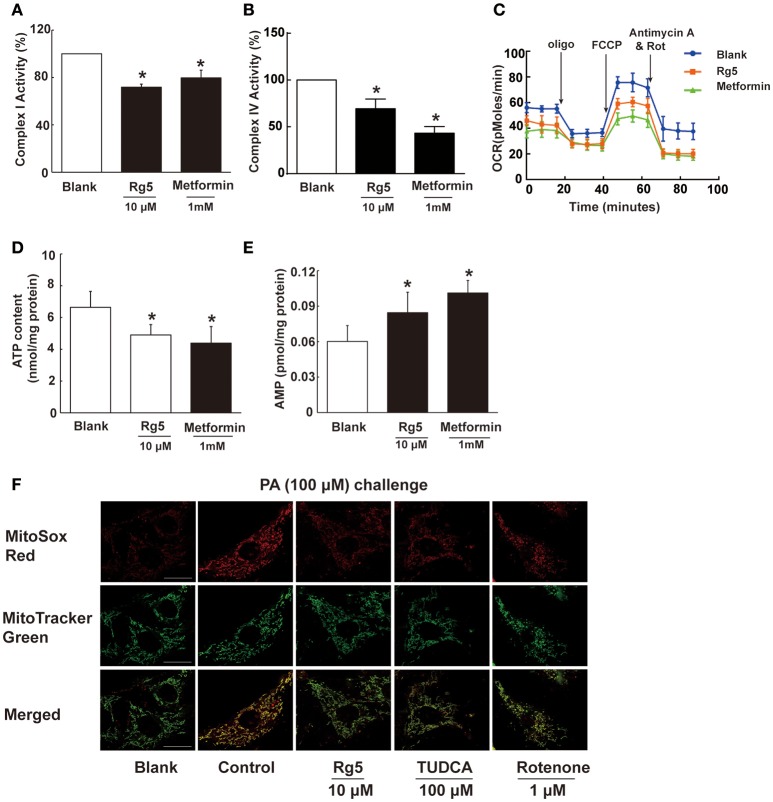
**Rg5 reduced cellular energy charge in differentiated adipocytes. (A,B)** Mitochondrial respiratory complex I and IV activity in differentiated adipocytes (*n* = 3). **(C)** Oxygen consumption ratio (OCR) in differentiated adipocytes (*n* = 4). **(D,E)** ATP and AMP contents in differentiated adipocytes (*n* = 6). **(F)** Mitochondrial ROS levels were measured by MitoSox Red (red) using confocal microscopy (*n* = 3). MitoSox Red was colocalized with MitoTracker Green. Scale bar, 20 μM. Data above were expressed as the mean ± SD. ^*^p < 0.05 *vs*. Blank. TUDCA, Tauroursodeoxycholic Acid.

To know the possible mechanism through which Rg5 prevented adipose hypoxia, we investigated the effects of Rg5 on cellular energy charge in adipocytes. Rg5 inhibited the activity of complex I and IV of the mitochondrial respiratory chain and reduced oxygen consumption ratio (OCR) in adipocytes (Figures 2A–C). Concordantly, Rg5 treatment effectively reduced intracellular ATP levels with increased AMP accumulation (Figures 2D,E). As a positive control, metformin exerted a similar effect in adipocytes. Meanwhile, we observed that Rg5 inhibited PA-induced mitochondrial ROS production in adipocytes (Figure 2F). Mitochondrial complex I inhibitor rotenone inhibited mitochondrial ROS generation, indicating that inhibition of mitochondrial complex I contributed to suppressing mitochondrial ROS production. These results indicate that Rg5 and metformin reduced cellular energy charge by mild inhibition of mitochondrial respiration in adipocytes.

### Rg5 suppressed ER stress in adipose tissue

**Figure 3:**
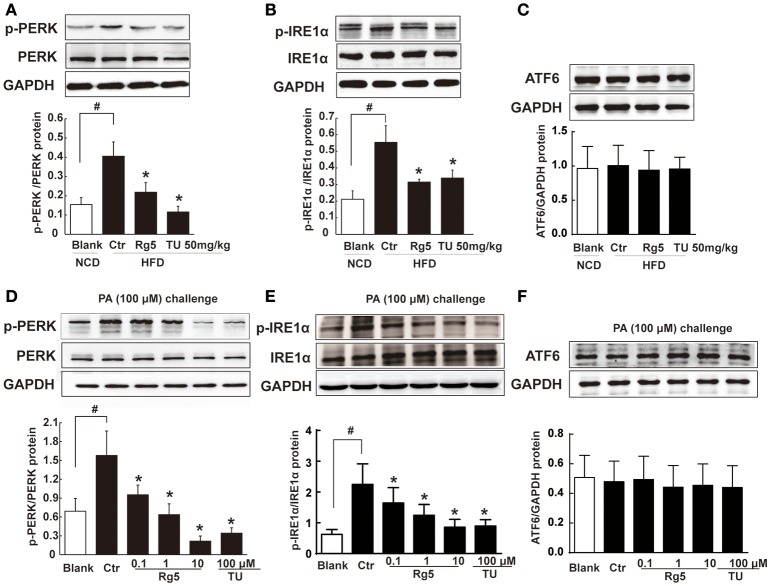
**Rg5 suppressed ER stress in adipose tissue. (A–C)** PERK, IRE1α phosphorylations (*n* = 3) and ATF6 protein expression (*n* = 4) in adipose tissue of HFD-fed mice. **(D–F)** PERK (*n* = 3), IRE1α (*n* = 4) phosphorylations and ATF6 (*n* = 4) protein expression in differentiated adipocytes incubated with palmitate (PA) for 8 h. The results were expressed as the mean ± SD. ^*^p < 0.05 *vs*. Control (Ctr), ^#^*p* < 0.05 *vs*. Blank. TU, Tauroursodeoxycholic Acid; oligo, oligomycin; rot, rotenone.

As a cellular stress, hypoxia could induce ER stress. HFD feeding increased the ER-localized protein sensor PERK and IRE1α phosphorylation in adipose tissue, while ATF6 expression was not affected (Figures 3A–C). Oral administration of Rg5 and TUDCA inhibited PERK and IRE1α activation by dephosphorylation without influence on ATF6 expression. Similarly, Rg5 inhibited PA-induced PERK and IRE1α phosphorylation in adipocytes without affecting ATF6 induction (Figures 3D–F). These results indicate the inhibitory effect of Rg5 on ER stress.

### Rg5 reduced succinate accumulation in adipose tissue

**Figure 4:**
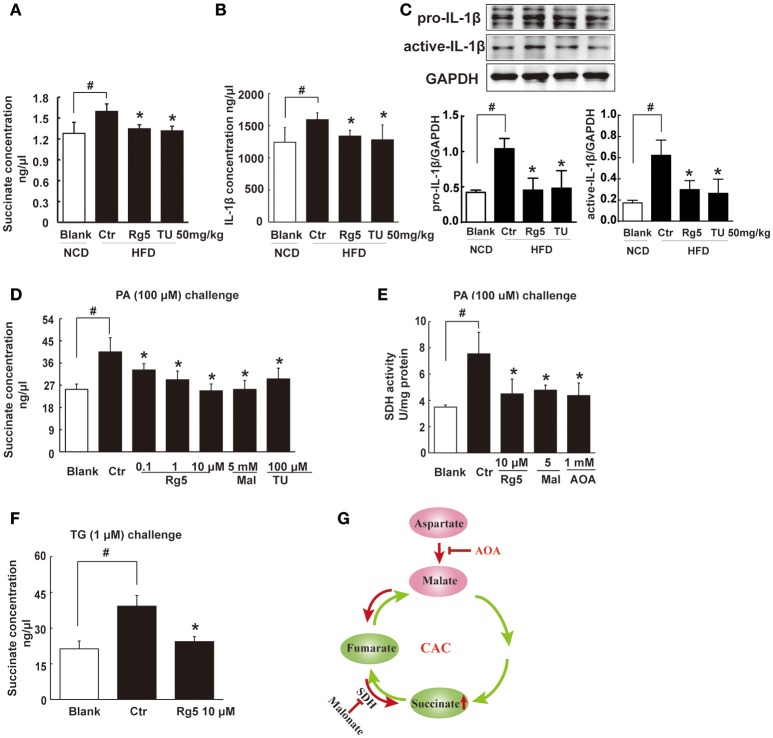
**Rg5 reduced succinate accumulation in adipose tissue. (A,B)** Succinate and IL-1β contents in adipose tissue of HFD mice (*n* = 6–8). **(C)** Protein expression of pro-IL-1β and active IL-1β in adipose tissue was determined by immunoblot analysis (*n* = 3). **(D,F)** Succinate accumulation in differentiated adipocytes treated with palmitate (PA) or thapsigargin (TG) for 8 h (*n* = 6). **(E)** SDH activity in differentiated adipocytes treated with PA for 8 h. **(G)** Summary of MAS pathway as potential drivers for the reversal of succinate accumulation. The results were expressed as the mean ± SD (*n* = 4). ^*^p < 0.05 *vs*. Control (Ctr), ^#^*p* < 0.05 *vs*. Blank. TU, Tauroursodeoxycholic Acid; AOA, aminooxyacetate; Mal, dimethyl malonate.

HFD feeding led to succinate accumulation in adipose tissue, and this alternation was prevented by Rg5 as well as TUDCA administration (Figure 4A). Meanwhile, Rg5 and TUDCA reduced IL-1β production and protein levels of pro-IL-1β and active IL-1β in adipose tissue (Figures 4B,C). Consistently, Rg5 as well as TUDCA reduced PA-induced succinate accumulation in differentiated adipocytes (Figure 4D). Interestingly, SDH inhibitor dimethyl malonate reduced succinate production, suggesting that succinate accumulation is the result of SDH activity reversal. To confirm this, we examined SDH activity in the presence of PA and found that Rg5 inhibited PA-induced SDH activation in adipocytes (Figure 4E). Aminooxyacetate (AOA), an inhibitor of aspartate aminotransferase in the malate/aspartate shuttle, inhibited SDH activity, indicating that malate/aspartate shuttle contributed to the reversal of SDH activity and subsequent succinate production (Figure 4E). TG causes ER stress by modulating Ca^2+^ levels and is usually used as an activator to induce ER stress. Rg5 reduced TG-induced succinate production, suggesting that the inhibitory effect of Rg5 contributed to reducing succinate production (Figure 4F). Taken together, these results indicated that Rg5 attenuated adipose hypoxia and then reduced succinate accumulation in adipose tissue. The proposed pathway for succinate production due to the reversal of SDH activity was shown in Figure 4G.

### Rg5 blocked cAMP/PKA signaling in adipose tissue

**Figure 5:**
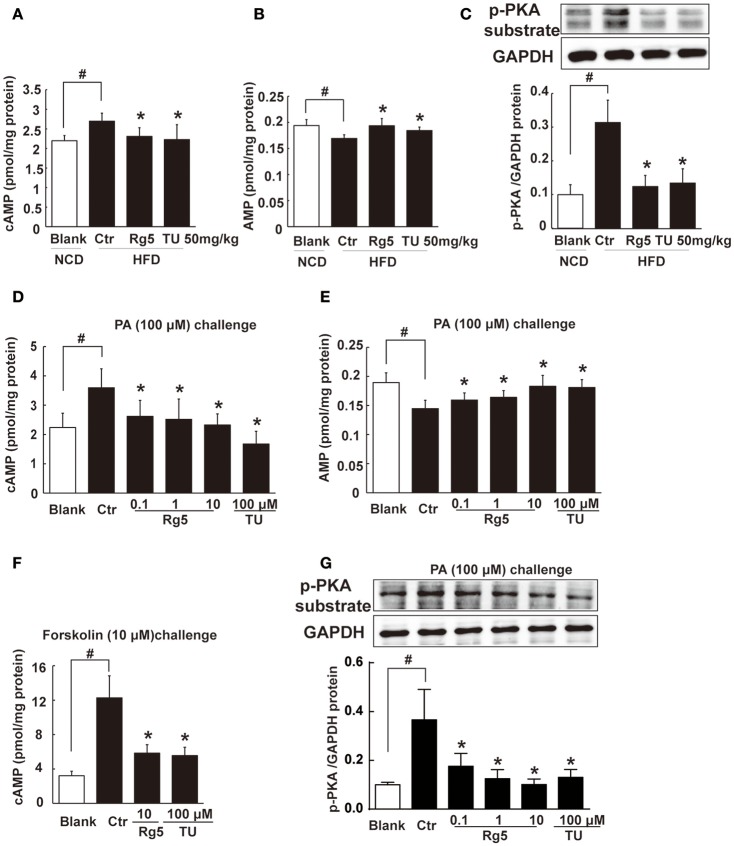
**Rg5 blocked cAMP/PKA signaling. (A,B)** cAMP and AMP contents in adipose tissue of HFD-fed mice (*n* = 8). **(C)** PKA phosphorylation in adipose tissue of HFD-fed mice (*n* = 3). **(D,E)** cAMP and AMP contents in differentiated adipocytes incubated with palmitate (PA) for 8 h (*n* = 6). **(F)** cAMP contents in differentiated adipocytes pretreated with agents for 4 h, and stimulated with forskolin for 0.5 h (*n* = 8). **(G)** PKA phosphorylation in differentiated adipocytes treated with PA for 8 h (*n* = 3). The results were expressed as the mean ± SD. ^*^p < 0.05 *vs*. Control (Ctr), ^#^*p* < 0.05 *vs*. Blank. TU, Tauroursodeoxycholic Acid.

HFD feeding increased cAMP accumulation (Figure 5A) with reduced AMP contents (Figure 5B) in adipose tissue, and these alternations were reversed by oral administration of Rg5 and TUDCA (Figures 5A,B). In line with the reduced cAMP production, adipose PKA 62 KDa substrate phosphorylation was attenuated in mice treated with Rg5 and TUDCA (Figure 5C).

Similar to the regulation of cAMP *in vivo*, Rg5 reduced PA-induced cAMP accumulation (Figure 5D) with increased AMP contents (Figure 5E) in adipocytes. As an activator of adenylate cyclase (AC), forskolin increased cAMP accumulation. Rg5 inhibited forskolin-induced cAMP accumulation (Figure 5F), suggesting that the reduced cAMP production might be related to the inhibition of AC activation by increased AMP. Consistent with reduced cAMP accumulation, Rg5 and TUDCA inhibited PA-induced PKA 62 KDa substrate phosphorylation in adipocytes (Figure 5G).

### Succinate increased cAMP accumulation in adipose tissue

**Figure 6:**
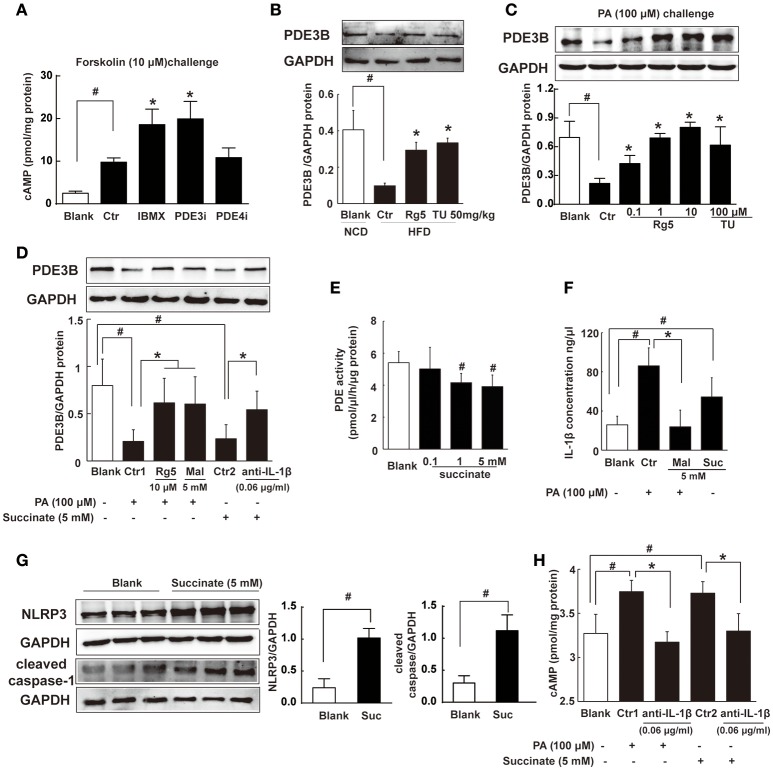
**Succinate increased cAMP accumulation. (A)** Differentiated adipocytes were incubated with 20 μM IBMX, 20 μM Cilostamide (PDE3Bi), or 50 μM Ro-20-1724 (PDE4i) for 0.5 h. Cells were treated with 10 μM forskolin for 0.5 h, lysed, and total cellular cAMP assayed. **(B,C)** PDE3B expression in adipose tissue of HFD-fed mice (*n* = 3) or in palmitate (PA)-treated adipocytes (*n* = 4) were determined with western blot. **(D)** PDE3B expression in PA- or succinate-treated differentiated adipocytes (*n* = 3). **(E)** PDE activity in differentiated adipocytes incubated with succinate for 8 h (*n* = 4). **(F)** IL-1β contents in differentiated adipocytes were determined with ELISA (*n* = 6). **(G)** NLRP3 and cleaved caspase-1 expression in differentiated adipocytes were determined with western blot (*n* = 3). **(H)** cAMP contents in adipocytes were determined with ELISA (*n* = 6). The results were expressed as the mean ± SD. ^*^p < 0.05 *vs*. Control (Ctr), ^#^*p* < 0.05 *vs*. Blank. TU, Tauroursodeoxycholic Acid; Mal, dimethyl malonate; Suc, dimethyl succinate.

Intracellular cAMP is synthesized by AC and can be degraded by phosphodiesterases (PDEs). In adipocytes, PDE inhibitor IBMX and PDE3 inhibitor cilostamide, but not PDE4 inhibitor Ro-20-1724, increased forskolin-induced cAMP production (Figure 6A). The result indicated that PDE3 is the predominant member of PDEs in adipocytes. PDE3A is more highly expressed in the cardiovascular system, while PDE3B is more highly expressed in adipocytes (Nilsson et al., 2006; Degerman et al., 2011). Thus, we observed PDE3B expression in adipose tissue of HFD-fed mice, and found that Rg5 and TUDCA preserved PDE3B induction (Figure 6B), indicative of the contribution to preventing cAMP accumulation.

Consistently, Rg5 and TUDCA protected PDE3B expression in adipocytes exposed to PA insult (Figure 6C). PA induced succinate formation due to the reversal of SDH activity, and therefore SDH inhibition reduced PA-mediated succinate production (Figure 4D). SDH inhibitor dimethyl malonate blocked the inhibitory effect of PA on PDE3B expression (Figure 6D), while dimethyl succinate, a cell-permeable derivative of succinate, reproduced PA action to inhibit PDE3B expression, suggesting that PA action was mediated by succinate (Figure 6D). Anti-IL-1β antibody diminished the inhibitory effect of succinate on PDE3B expression (Figure 6D). Meanwhile, we examined the effect of succinate on PDEs activity in differentiated adipocytes and found that dimethyl succinate exhibited inhibitory effect on PDEs activity (Figure 6E). PDE3 activity was inhibited by PDE inhibitor IBMX, while AC activation could further increase cAMP content. However, succinate failed to change the elevated levels of cAMP in the presence of IBMX, indicating that it did not influence AC activity (Supplementary Figure 1).

We hypothesized that succinate mediated PA action to suppress PDE3B expression in a manner dependent on IL-1β. To confirmed this, we observed IL-1β production in adipocytes and found that both PA and succinate increased IL-1β production (Figure 6F). SDH inhibitor dimethyl malonate diminished the enhancing effect of PA on IL-1β production (Figure 6F), suggesting the increased IL-1β production by PA depended on succinate, at least in part. IL-1β maturation is mediated through NLRP3 inflammasome activation. Succinate increased NLRP3 and cleaved caspase-1 expression, indicating the activation of NLRP3 inflammation (Figure 6G). Succinate induced cAMP production and co-treatment with anti-IL-1β antibody diminished the enhanced effects of PA and succinate on cAMP production (Figure 6H). These results provided evidence to support our hypothesis that succinate was a mediator in PA action and increased cAMP accumulation *via* NLRP3 inflammasome activation.

### Rg5 inhibited FFAs release and inflammation in adipose tissue

**Figure 7:**
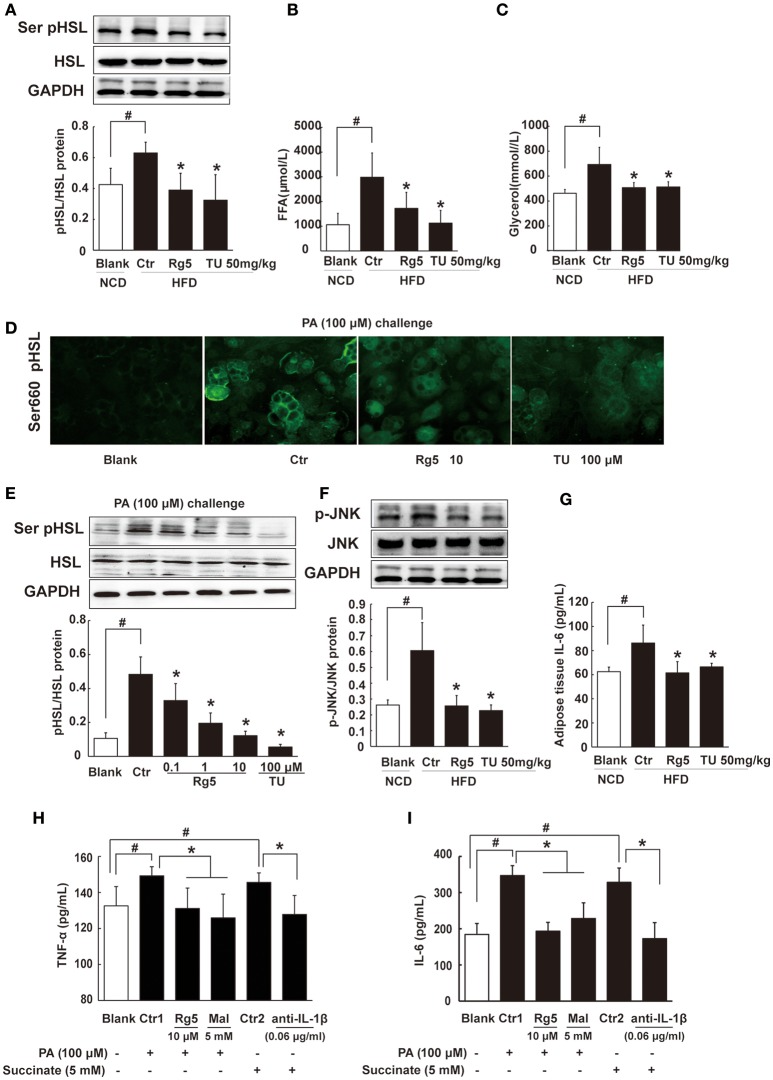
**Rg5 inhibited FFAs release and inflammation in adipose tissue. (A)** HSL phosphorylation in adipose tissue of HFD-fed mice (*n* = 3). **(B,C)** FFAs, Glycerol release from adipose tissue of HFD-fed mice (*n* = 6–8). **(D,E)** HSL recruitment and phosphorylation in adipocytes treated with palmitate (PA) (*n* = 3) for 8 h. **(F,G)** JNK expression (*n* = 3) and IL-6 (*n* = 6) contents in adipose tissue of HFD mice. **(H,I)** TNF-α and IL-6 contents in adipocytes (*n* = 6). GAPDH band was duplicated for **(A,F)** as a result of the experiments having been performed at the same time. Data were expressed as the mean ± SD. ^*^p < 0.05 *vs*. Control (Ctr), ^#^*p* < 0.05 *vs*. Blank. TU, Tauroursodeoxycholic Acid; Mal, dimethyl malonate.

Lipolysis is initiated from cAMP/PKA signaling. Consistent with reduced cAMP accumulation and suppressed PKA substrate phosphorylation, oral administration of Rg5 and TUDCA suppressed hormone-sensitive lipase (HSL) phosphorylation, and then reduced FFAs and glycerol release from adipose tissue in HFD-fed mice (Figures 7A–C), suggesting their inhibitory effects on lipolysis. Similar to the regulation *in vivo*, Rg5 and TUDCA also inhibited HSL translocation to the surface of the lipid droplets, prevented PA-induced HSL phosphorylation (Figures 7D,E) and reduced FFAs and glycerol release from differentiated adipocytes (Supplementary Figure 2). Rg5 and TUDCA attenuated JNK phosphorylation and reduced IL-6 production in the adipose tissue of HFD-fed mice, indicating their anti-inflammatory activity (Figures 7F,G). In adipocytes, Rg5 also effectively reduced PA-induced TNF-α and IL-6 production (Figures 7H,I). SDH inhibitor dimethyl malonate inhibited PA-induced TNF-α and IL-6 production and anti-IL-1β antibody diminished the enhancing effects of succinate on TNF-α and IL-6 production (Figures 7H,I), indicating that succinate-derived IL-1β was paramount in inflammation.

### Rg5 improved glucose tolerance in HFD-fed mice

HFD feeding led to elevated levels of FFAs, glycerol, and total cholesterol in the blood, while the level of triglyceride remained unchanged. Oral administration of Rg5 reduced the elevated levels of blood FFAs and glycerol without affecting other metabolic parameters, including food intake and body weight gain in mice (Supplementary Figure 3).

**Figure 8:**
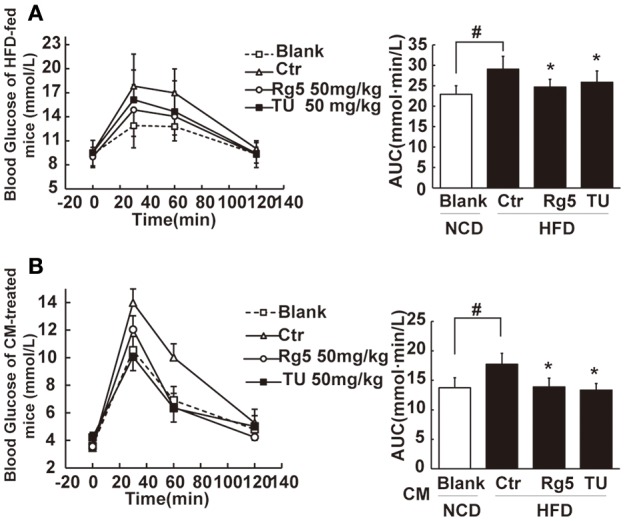
**Rg5 improved glucose tolerance in HFD-fed mice. (A)** Oral glucose tolerance in HFD mice. **(B)** Oral glucose tolerance in normal mice treated with CM. Data were expressed as the mean ± SD (*n* = 10). ^*^p < 0.05 *vs*. Control (Ctr), ^#^*p* < 0.05 *vs*. Blank. TU, Tauroursodeoxycholic Acid.

Oral administration of Rg5 increased glucose disposal after glucose load, leading to reduction in the total glucose (Figure 8A). To confirm the beneficial effect was associated with the regulation of adipose function, we prepared conditioned medium (CM) by incubation of the adipose tissue of HFD-fed mice, and then intraperitoneally injected normal mice by with CM. We observed that the glucose tolerance was impaired in CM-treated mice. The glucose intolerance was prevented when the CM was derived from Rg5 or TUDCA-treated mice during HFD feeding (Figure 8B). These results indicated that the beneficial regulation of adipose function by Rg5 contributed to improving glucose homeostasis.

### Rg5 reduced lipid deposits and improved insulin signaling in the muscle

**Figure 9:**
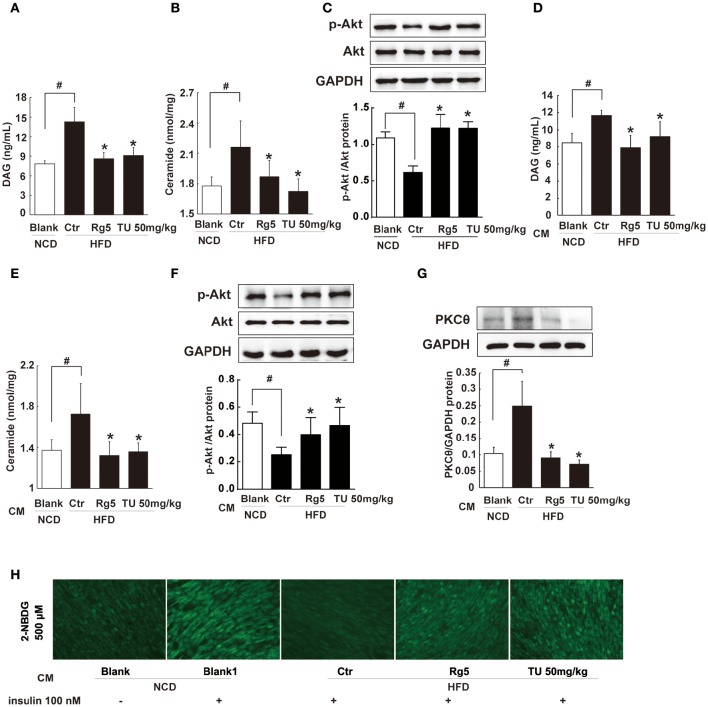
**Rg5 reduced DAG and ceramide contents and improved insulin signaling in the muscle. (A,B)** DAG and ceramide contents in the muscle of HFD mice (*n* = 6–8). **(C)** Akt phosphorylation in response to glucose load in the muscle of HFD mice (western blot, *n* = 4). **(D–F)** Normal mice were injected with CM, DAG, ceramide contents (*n* = 6–8) and glucose load-induced Akt phosphorylation (*n* = 5) in the muscle were determined with ELISA or western blot. **(G,H)** Differentiated C2C12 myotubes were incubated with CM, and membrane PKCθ and glucose uptake were examined. Data were expressed as the mean ± SD (*n* = 3). ^*^p < 0.05 *vs*. Control (Ctr), ^#^*p* < 0.05 *vs*. Blank. TU, Tauroursodeoxycholic Acid.

To disclose the potential relation between the increased circulating FFAs and insulin resistance, we investigated lipid deposits and insulin signaling in skeletal muscle. HFD feeding increased DAG and ceramides contents in the muscle (Figures 9A,B) and attenuated Akt phosphorylation in response to glucose load (Figure 9C), indicating the presence of insulin resistance. Oral administration of Rg5 or TUDCA reduced DAG and ceramides contents and improved insulin signaling by normalizing Akt phosphorylation in the muscle.

To explore the contribution of trafficking FFAs to muscle insulin resistance, we treated normal mice with HFD fat-derived CM, and found that CM challenge increased DAG and ceramides accumulation in the muscle with attenuated Akt phosphorylation (Figures 9D–F). When the CM was prepared from the adipose tissue of Rg5 or TUDCA-treated mice in HFD-feeding, these alternations were prevented.

Incubation of muscle cells with HFD fat-derived CM promoted PKCθ translocation to the cell membrane and inhibited insulin-mediated glucose uptake in muscle cells, but these changes were prevented when the CM was prepared from Rg5-treated mice in the course of HFD feeding (Figures 9G,H). These data suggest that Rg5 inhibited lipolysis in adipose tissue and prevented insulin resistance in muscle by reducing lipid deposits.

## Discussion

Succinate is a metabolic intermediate whereas in hypoxic adipose tissue it acted as a metabolic signaling molecule mediating PA action to induce inflammation-associated lipolysis. Rg5 ameliorated succinate-associated adipose dysfunction, inhibited lipolysis, and prevented muscle insulin resistance, providing new insight into the role of Rg5 in the regulation of lipid and glucose homeostasis.

In the present study, we showed that short-term HFD feeding induced hypoxia in adipose tissue of mice. These results indicate that adipose hypoxia is an early event in adipose dysfunction and provide new insight into the impact of lipotoxicity. To know the possible mechanism through which Rg5 prevented adipose hypoxia, we investigated the effects of Rg5 on cellular energy charge, a process associated with oxygen consumption. Rg5 reduced intracellular ATP/AMP ratio, resulting in adipocytes of low energy charge. The inhibitory effect on mitochondrial complex I and IV activity should be the main cause for the reduced energy charge. Since oxygen is consumed at complex IV, the inhibitory effect of Rg5 on mitochondrial complex IV should contribute to preventing adipose hypoxia by reducing oxygen consumption. Rg5 reduced oxygen consumption in adipocytes, providing evidence to support our hypothesis. As an anti-diabetic agent, metformin reduces cellular energy by inhibiting mitochondrial complex I (Foretz et al., 2010), and this action is proposed to be instrumental in the prevention of hypoxia in the kidney (Takiyama et al., 2011). Similarly, we observed that metformin prevented hypoxia in adipocytes exposed to 1% O_2_ (Figure 1F). Based on above-mentioned evidence, it is reasonable to believe that Rg5 prevented adipose hypoxia by reducing cellular energy charge.

Although ER stress is associated with ischemic injury in heart and brain (Thuerauf et al., 2006; Xin et al., 2014), its action in hypoxic adipose tissue is little known. TUDCA is a chemical chaperone with the ability to suppress ER stress (Ozcan et al., 2006). HFD feeding as well as direct PA challenge decreased cellular oxygen tension with ER stress, while ER stress inhibitor TUDCA attenuated cellular hypoxia and prevented HIF-1α accumulation, indicating that ER stress was involved in adipose hypoxia. HFD feeding or PA challenge induced PERK and IRE1α branches activation without alternation of ATF6. These results are also consistent with published studies, which show that PERK and IRE1α activation is involved in adipose dysfunction (da Luz et al., 2011; Tsutsumi et al., 2011; Li et al., 2015). Rg5 attenuated hypoxia in adipocytes, and this regulation should contribute to reducing stress burden and HIF-1α induction. Therefore, our work suggests that cellular energy limitation might be a possible way to prevent ER stress in adipose dysfunction.

Adipose lipolysis cascades are initiated by cAMP/PKA signaling. As a second messenger in cellular signal transduction, cAMP mediates cellular response to stress. The levels of cAMP are regulated by AC and PDEs: AC increases cAMP production while the accumulated cAMP is degraded by PDEs. More than 40 years ago, adenosine nucleosides were found to inhibit AC activity (Fain et al., 1972). Biguanides and metformin are shown to suppress hepatic AC activity and reduce cAMP concentration by elevating the levels of AMP (Miller et al., 2013). Rg5 reduced cellular energy charge with increased AMP production, and the elevated levels of AMP could contribute to the suppression of AC activity and cAMP production. Forskolin binds to and stimulates AC activity directly without the regulation of G protein. Rg5 reduced forskolin-induced cAMP production, further indicating that it inhibited AC activity *via* reducing cellular energy charge, a regulation independent of G protein.

Although the increased AMP content was able to prevent cAMP production by inhibition of AC activity, this regulation could not explain the effect of Rg5 on the preserved PDE3B induction. Interestingly, we observed increased succinate accumulation in the adipose tissue of HFD-fed mice. Succinate is an intermediate metabolite of the CAC and plays a crucial role in energy metabolism. In the CAC, the conventional direction of SDH is for the formation of fumarate from succinate, whereas during anaerobic metabolism SDH might act in reverse to reduce fumarate to succinate (Taegtmeyer, 1978; Chouchani et al., 2014). In the present study, PA challenge induced hypoxia in adipocytes and increased succinate production with SDH activation. These results indicate the converse role of SDH in the hypoxic adipocytes, since activation of SDH operating in its conventional direction should decrease succinate accumulation due to the formation of fumarate. Inhibition of SDH activity with dimethyl malonate reduced succinate accumulation (Figure 4D), further confirming the converse role of SDH in succinate production. As an inhibitor of malate/aspartate shuttle, AOA inhibited SDH activity (Figure 4E), indicating that malate/aspartate shuttle inhibition blocked the supply of fumarate for succinate formation (Chouchani et al., 2014). Different from ischemic succinate accumulation in the heart (Chouchani et al., 2014), our work showed that saturated fatty acid induces succinate production *via* increased mitochondrial energy charge. Hypoxia induced ER stress and TG increased succinate accumulation (Figure 4F), suggesting that ER stress exacerbated metabolic disorders in hypoxic adipose tissue.

Adipose dysfunction is tightly associated with inflammation. A recent study suggests that cellular succinate production induced IL-1β expression in macrophages (Tannahill et al., 2013). Consistent with this, we found that succinate promoted IL-1β production in hypoxic adipocytes with NLRP3 inflammasome activation. The NLRP3 inflammasome is a molecular platform composed of NLRP3, the adaptor protein ASC and caspase-1, and its action is to promote IL-1β maturation and secretion through caspase-1 cleavage (Schroder and Tschopp, 2010). Succinate directly increased NLRP3 and cleaved caspase-1 induction, indicating NLRP3 inflammasome activation. Therefore, our work indicates that succinate-induced IL-1β secretion should be a result of NLRP3 inflammasome activation. Consistent with a recent published study, the replacement of saturated fatty acid for monounsaturated fatty acid in high fat diet could ameliorate IL-1β-mediated adipose dysfunction and insulin resistance in mice, highlighting the special role of saturated acid in NLRP3 inflammasome activation (Finucane et al., 2015). Succinate impaired PDE3B expression and inhibited PDEs activity (Figures 6D,E), while neutralization of IL-1β with anti-IL-1β antibody diminished its inhibitory effect on PDE3B activation (Figure 6D), indicating that IL-1β was involved in the impairment of PDE3B expression. Succinate-mediated cAMP production was blocked by treatment with anti-IL-1β antibody (Figure 6H), providing evidence to prove the speculation. Because succinate was derived from PA-induced metabolic dysregulation, these results indicated that succinate mediated PA action to induce cAMP accumulation in an inflammation-dependent way. In fact, inflammatory signaling and pro-inflammatory cytokines have been shown to increase cAMP accumulation by downregulation of PDE activity in adipose tissue and liver (Zhang et al., 2002; Ke et al., 2015). Moreover, our work suggests the possibility that succinate-derived IL-1β might act as an initiator of inflammation in adipose dysfunction. Indeed, we observed that TNF-α and IL-6 produciton were blocked by neutralizing IL-1β with IL-1β anti-body. It is well-established that obesity is a low grade inflammation and saturated fatty acid palmitate could act as a ligand for toll-like receptor 4 (TLR4) to induce inflammation in adipocytes (Davis et al., 2009). Consistent with the published studies, ginsenosides inhibited inflammation *via* suppression of TLR4 activation in macrophages (Kim et al., 2012; Lee I. A. et al., 2012) our work presented another regulation for Rg5 to ameliorate inflammation-mediated adipose dysfunction. Although our work indicated that succinate acted as a metabolic signaling linking altered metabolism with inflammation in adipose tissue, the functional interaction with TLR4-mediated adipose dysfunction is unknown. Succinate mediates lipopolysaccharide-induced inflammation in macrophages, suggesting the possibility in adipocytes (Tannahill et al., 2013).

Rg5 inhibited cAMP/PKA activation and reduced lipolysis, and this action should prevent lipid deposits and insulin signaling impairment in muscle. We prepared conditioned medium (CM) from the adipose tissue of HFD-fed mice to induce DAG accumulation and PKCθ translocation in muscle, establishing the functional connection between adipose lipolysis and muscle insulin resistance. DAG/PKCθ activation is shown to induce insulin resistance by impairing insulin signaling in muscle (Szendroedi et al., 2014). DAG may activate different isoforms of PKC to impair insulin signaling (Madani et al., 2001). For example, in hepatocytes, DAG could promote PKCϵ translocation and induce hepatic insulin resistance (Samuel et al., 2004). The different DAG species or competitive effects of specific DAG species might be the reason for the activation of different PKC isoforms in special cells (Madani et al., 2001). In addition to DAG, increased FFAs supply can raise intracellular ceramides, which induce inflammation-associated insulin resistance by impairing insulin-mediated Akt activation in muscle (Chaurasia and Summers, 2015; Ritter et al., 2015). Oral administration of Rg5 inhibited lipolysis in HFD-fed mice and prevented lipid deposits and PKCθ activation in muscle, and thus improved insulin signaling and promoted glucose uptake in muscle cells. These results indicated that inhibition of adipose lipolysis could prevent muscle insulin resistance.

**Figure 10:**
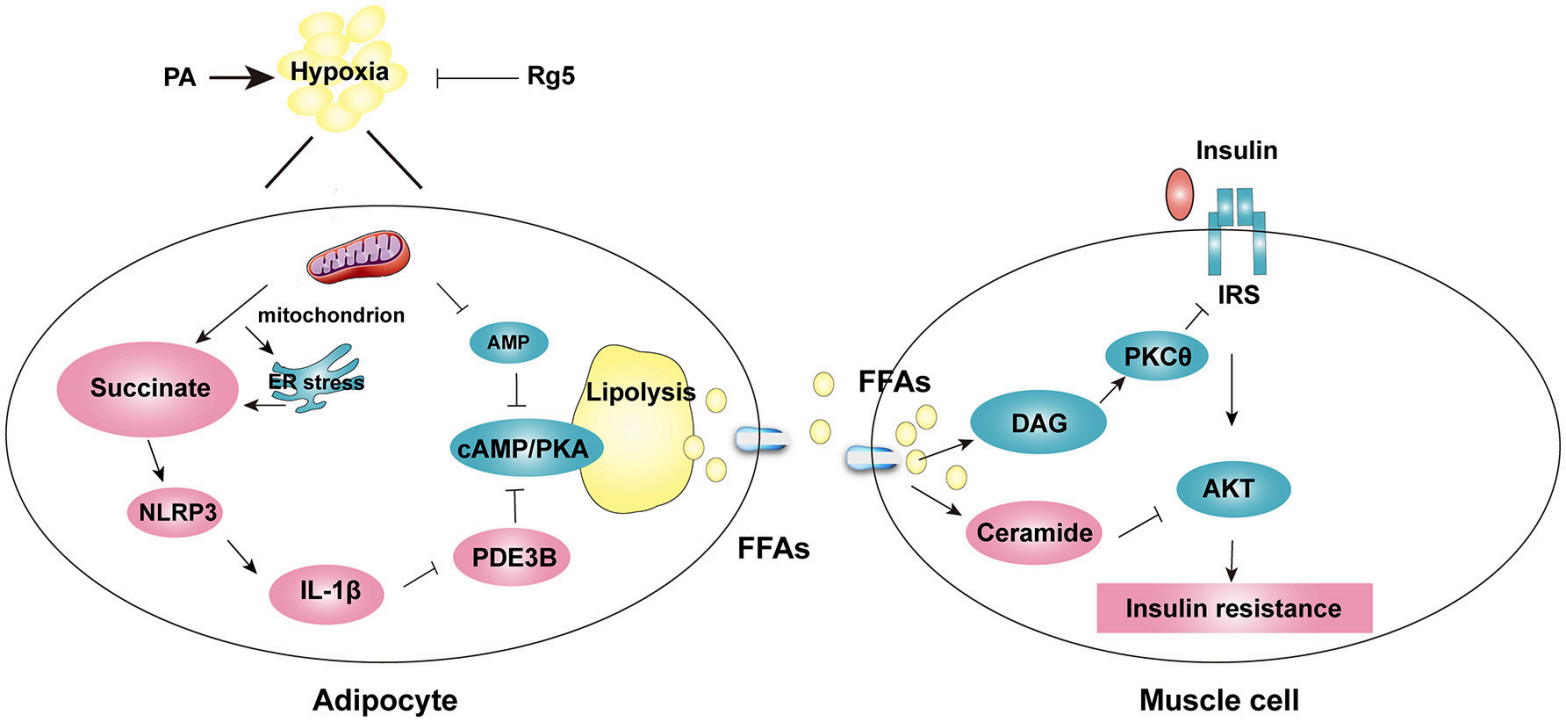
**The proposed working pathway for ginsenoside Rg5 action in hypoxic adipose tissue**. Saturated fatty acid PA induces hypoxia with ER stress in adipose tissue, and then increases succinate formation. Succinate promotes IL-1β production through NLRP3 inflammasome activation, and then induces cAMP/PKA signaling by inactivation of PDE3B, leading to lipolysis. PA also decreases AMP concentration, and then increases cAMP accumulation. The release of FFAs increases cytosolic DAG, PKC translocation, ceramide accumulation and inhibits insulin-mediated Akt phosphorylation, thus leading to insulin resistance in muscle. Rg5 inhibits lipolysis by suppression of succinate accumulation in hypoxic adipocytes and thus prevents insulin resistance in muscle.

Our work indicates that ER stress, inflammation, and lipolysis simultaneously occurred in adipose dysfunction, and succinate as a key mediator linking these events. Ginsenoside Rg5 inhibited succinate-associated lipolysis *via* reducing cellular energy charge, and effectively prevented insulin resistance in muscle by reducing lipid deposits. The proposed mechanistic pathway was shown in Figure 10. Although the beneficial effects of ginsenosides on adipose function and insulin sensitivity have been documented (Yu et al., 2015; Chen et al., 2016), we provides new insight into the cellular basis for Rg5 in the regulation of lipid and glucose homeostasis. Moreover, our work suggests that blocking FFAs trafficking from adipose to muscle might be a potential therapeutic strategy for the prevention of insulin resistance.

## Author contributions

LQ, PL designed the research. NX performed experiments, analyzed data, and drafted the manuscript. LY, YY, JL, and LW collected data and reviewed the manuscript. LQ edited the manuscript. BL and KL contributed to the discussion and review of the manuscript. All authors approved the final version of the paper.

## Funding

This work was supported in part by the National Natural Science Foundation of China (No. 81421005, 81603353, 91639115), National Excellent Doctoral Dissertation of PR China (No. 201278), and Natural Science Foundation of Jiangsu Province (BK20160762).

### Conflict of interest statement

The authors declare that the research was conducted in the absence of any commercial or financial relationships that could be construed as a potential conflict of interest.
