# Supplementary figures and images for: Ginsenoside Rg5 Inhibits Succinate-Associated Lipolysis in Adipose Tissue and Prevents Muscle Insulin Resistance
Source: Front Pharmacol. 2017 Feb 14;8:43. doi: 10.3389/fphar.2017.00043 (PMC5306250; doi:10.3389/fphar.2017.00043)

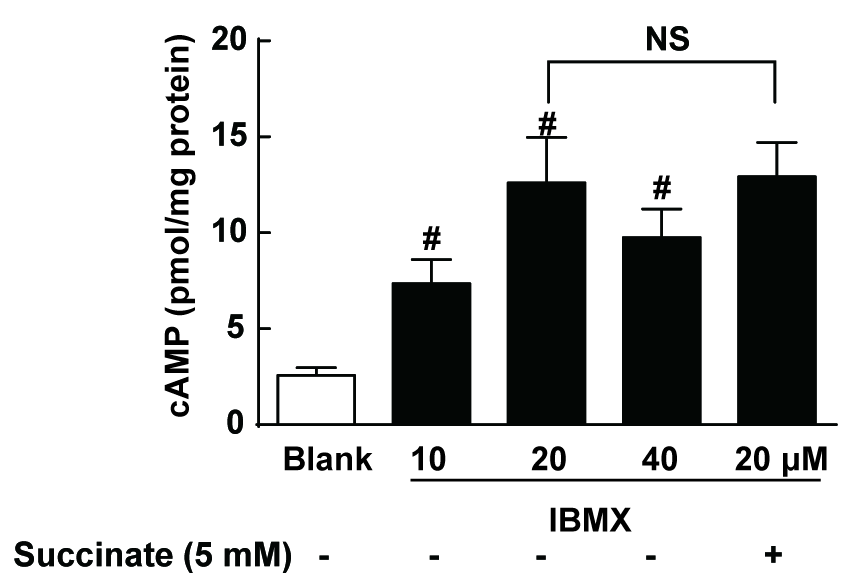

Supplement: Supplementary file 1 [file Image1.TIF]

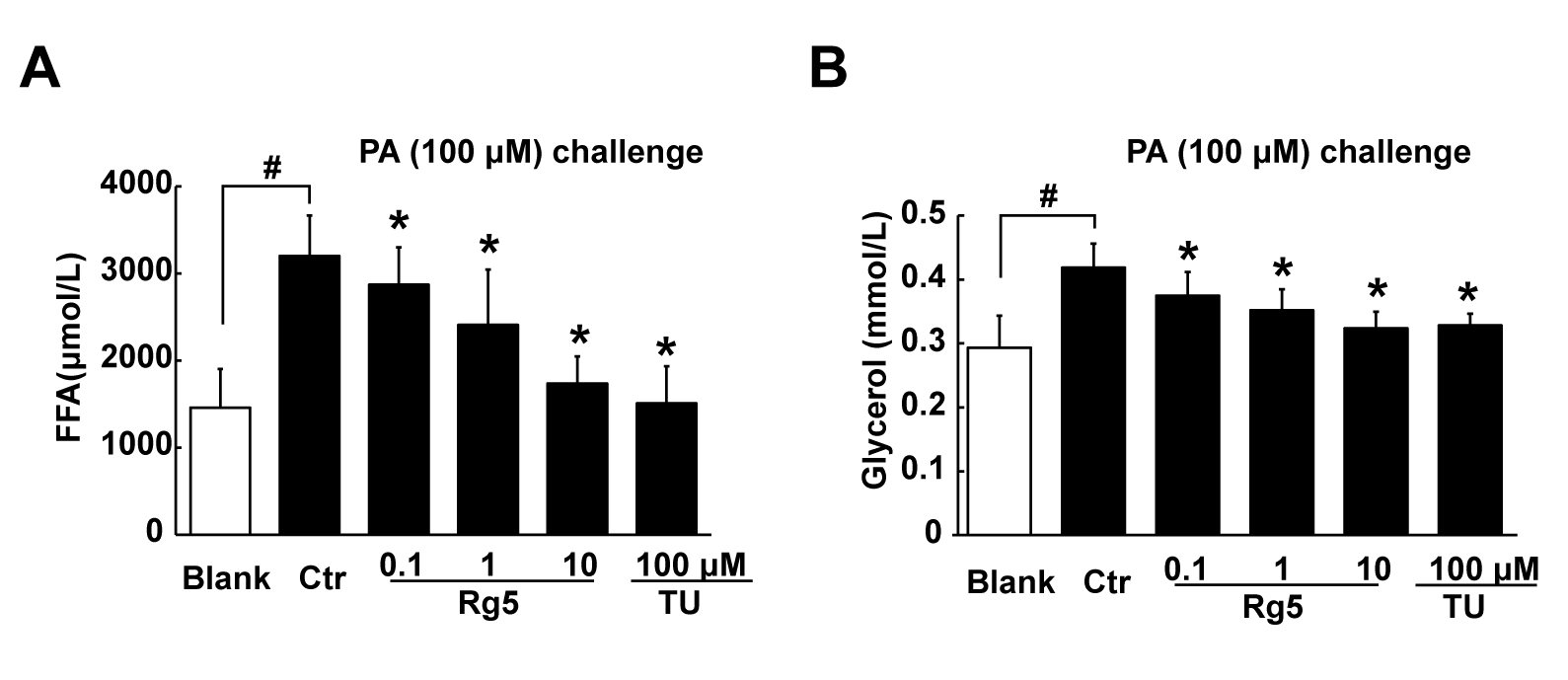

Supplement: Supplementary file 2 [file Image2.TIF]

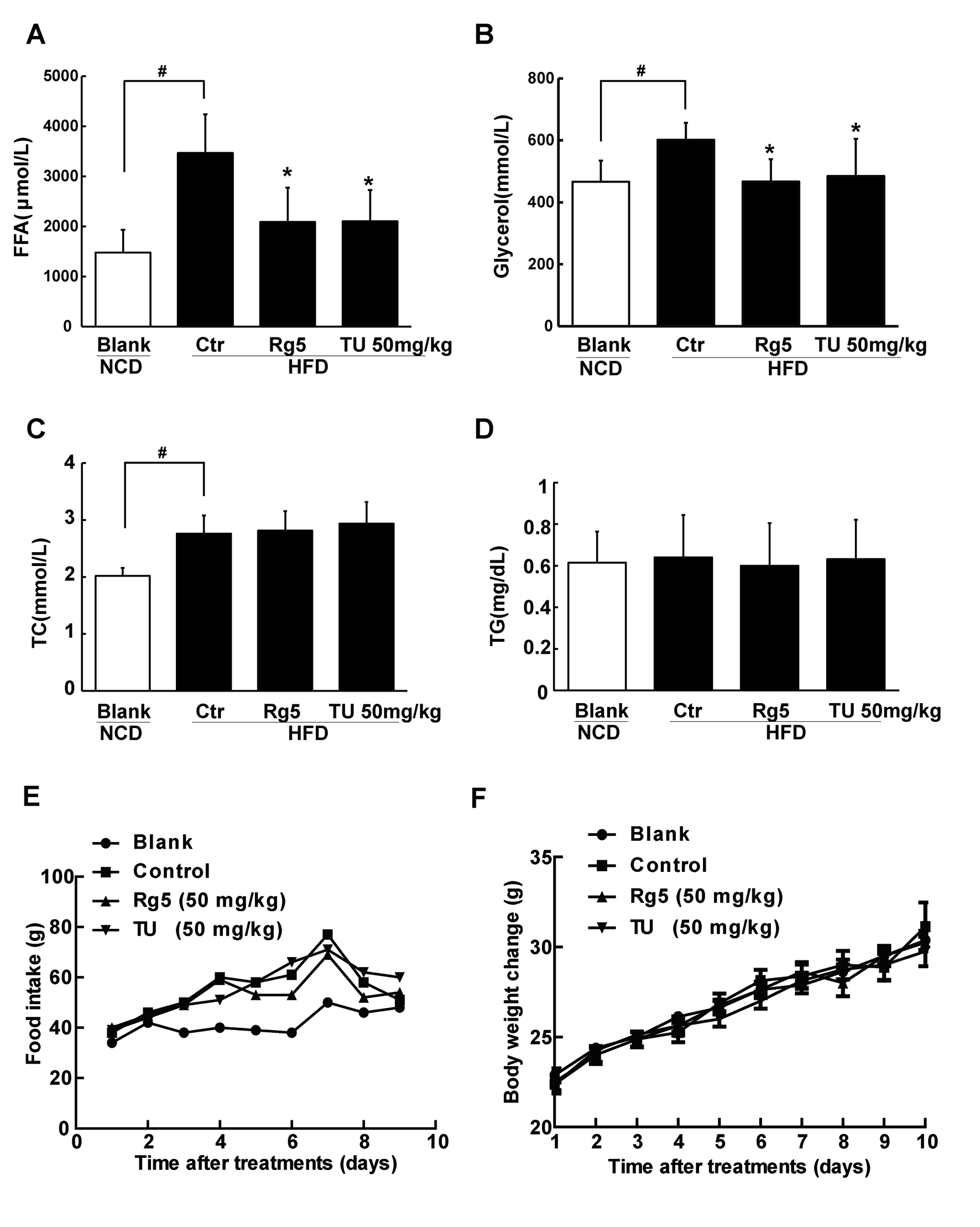

Supplement: Supplementary file 3 [file Image3.TIF]
