# Supplementary material for: Ginsenoside Rg5 Inhibits Succinate-Associated Lipolysis in Adipose Tissue and Prevents Muscle Insulin Resistance
Source: Front Pharmacol. 2017 Feb 14;8:43. doi: 10.3389/fphar.2017.00043 (PMC5306250; doi:10.3389/fphar.2017.00043)
Supplement: Supplementary file 4 [file DataSheet1.DOCX]

**Supplementary Figure 1.** Adipocytes were incubated with IBMX (10, 20 or 40 μM), with or without 5 mM succinate for 2 h. Cells were lysed, and total cellular cAMP assayed. Data were expressed as the mean ± SD (*n*=6). ^#^*p* < 0.05 *vs.* Blank, ^NS^*p* > 0.05 *vs.* the indicated treatment.

**Supplementary Figure 2.** Rg5 inhibited lipolysis in adipocytes. (**A, B**): FFAs and glycerol release from adipocytes treated with PA (*n*=6). Data were expressed as the mean ± SD. **p* < 0.05 *vs.* Control (Ctr), ^#^*p* < 0.05 *vs.* Blank.

**Supplementary Figure 3.** Rg5 reduced blood FFAs and glycerol in high-fat fed mice. (**A-D**): Blood was collected from orbital sinus for FFAs, glycerol, total cholesterol (TC) and triglyceride (TG) used commercial kits. (**E, F**): Food intake and body weight evaluation of mice treated with Rg5 every day. Data were expressed as the mean ± SD (*n*=6-8). **p* < 0.05 *vs.* Control (Ctr), ^#^*p* < 0.05 *vs.* Blank. (NCD, normal control food).
